# Characterisation of the Muscle Protein Synthetic Response to Resistance Exercise in Healthy Adults: A Systematic Review and Exploratory Meta-Analysis

**DOI:** 10.1155/2024/3184356

**Published:** 2024-04-30

**Authors:** Robert W. Davies, Arthur E. Lynch, Uttam Kumar, Philip M. Jakeman

**Affiliations:** ^1^Chester Medical School, University of Chester, Shrewsbury, UK; ^2^Department of Physical Education and Sport Sciences, University of Limerick, Limerick, Ireland; ^3^Health Research Institute, University of Limerick, Limerick, Ireland

## Abstract

**Methods:**

Five electronic databases (PubMed (Medline), Web of Science, Embase, Sport Discus, and Cochrane Library) were searched for controlled trials that assessed the MPS response to RE in healthy, adult humans, postabsorptive state. Individual study and random-effects meta-analysis arewere used to inform the effects of RE and covariates on MPS. Results from 79 controlled trials with 237 participants were analysed.

**Results:**

Analysis of the pooled effects revealed robust increases in MPS following RE (weighted mean difference (WMD): 0.032% h^−1^, 95% CI: [0.024, 0.041] % h^−1^, *I*^2^ = 92%, *k* = 37, *P* < 0.001). However, the magnitude of the increase in MPS was lower in older adults (>50 y: WMD: 0.015% h^−1^, 95% CI: [0.007, 0.022] % h^−1^, *I*^2^ = 76%, *k* = 12, *P* = 0.002) compared to younger adults (<35 y: WMD: 0.041% h^−1^, 95% CI: [0.030, 0.052] % h^−1^, *I*^2^ = 88%, *k* = 25, *P* < 0.001). Individual studies have reported that the temporal proximity of the RE, muscle group, muscle protein fraction, RE training experience, and the loading parameters of the RE (i.e., intensity, workload, and effort) appeared to affect the MPS response to RE, whereas sex or type of muscle contraction does not.

**Conclusion:**

A single bout of RE can sustain measurable increases in postabsorptive MPS soon after RE cessation and up to 48 h post-RE. However, there is substantial heterogeneity in the magnitude and time course of the MPS response between trials, which appears to be influenced by participants' age and/or the loading parameters of the RE itself.

## 1. Introduction

Healthy human skeletal muscle tissue demonstrates remarkable plasticity, rapidly adapting to nutritional, contractile in/activity, and micro/environmental changes [1]. The fractional *rate* of muscle protein synthesis (MPS) (i.e., the rate at which amino acids are incorporated into new skeletal muscle proteins) is considered the principal determinant of net protein balance and the driving force underpinning the adaptive responses within the muscle (e.g., remodelling, repair, regeneration, and/or growth) to repeated high-force contractile activity, such as resistance exercise (RE) [2, 3].

MPS is quantitatively assessed as an average over short (i.e., hourly) or long (i.e., days, weeks, and months) time durations [4–11], via the precursor-product method. This method involves the administration of naturally occurring stable isotopically labelled amino acids combined with sampling of biological fluids (e.g., plasma and/or saliva) and skeletal muscle tissue (via percutaneous biopsy), and mass spectrometry (MS), which are used to determine the rate at which the labelled amino acids are incorporated into skeletal muscle protein over a predefined period of time [2, 3]. This approach of quantifying MPS requires infusion of isotopically labelled amino acids and/or amino acid precursors (e.g., [1-^13^C] leucine, [1-^13^C] *α*-ketoisocaproate, [^15^N] proline, [1-^13^C] valine, [^2^H_2_] phenylalanine, or [ring-13C_6_] phenylalanine), intra-venous/arterial cannulation, and multiple biopsies [2, 3, 12]. Consequently, experimental trials are *de facto* short (i.e., hours) and conducted under tightly controlled laboratory conditions, when determined over longer periods (i.e., days); usually, the oral dosing of deuterium oxide (D2O) is applied [9–11, 13].

Feeding (specifically ingestion of high-quality protein or essential amino acids (EAA)) and RE can independently (and synergistically) stimulate fold-increases in MPS in a dose-dependent manner [2, 12, 14]. However, unlike the short-lived EAA-induced increase in MPS, which returns to baseline levels once the muscle full limit is reached (typically 2 to 8 h [15, 16]), the RE-induced increases in MPS can be sustained >24 h post-RE under postabsorptive conditions [17]—emphasising the importance of the contractile regulation of MPS. However, the variation of the MPS response to RE in different populations to different RE and/or experimental protocols is less clear. Consequently, information pertaining to the regulation of RE-induced changes in MPS may have implications for growth, development, and/or preservation of skeletal muscle tissue in humans along with its corollaries (e.g., metabolic health and physical function across the adult age span) [18, 19].

Moreover, there is an extensive body of research on the regulation of the MPS response to RE (e.g., nutritional/nutraceutical, pharmacological, or environmental interventions [5, 11, 20–26]). The absence of definitive knowledge pertaining to “normative” or “typical” MPS response to RE and/or a standardised “model” of RE limits external validity, context, and certainty of findings, i.e., ability to compare results or demonstrate consistency across trials. Therefore, the provision of empirical data outlining the magnitude, variation, and moderators of the MPS response to RE will afford researchers the ability to better plan/power new studies, contextualise their findings, and more accurately/easily discern the impact of their interventions.

Over the last 30 years, the MPS response to RE in humans has been subject to a significant amount of scientific research across a range of participant demographics, RE interventions, and experimental, methodological, and analytical approaches [2, 3, 12]. Indeed, carefully controlled, laboratory-based measures of MPS have allowed the direct effect of RE to be analysed and provided crucial information regarding the acute remodelling, repair, and regenerative response to RE [27–32]. However, to our knowledge, this body of research has not been systematically reviewed and/or quantitatively assessed *in toto*. Despite potential challenges associated with comparing findings across independent experimental trials, (e.g., differing experimental designs, methodologies and procedures, participant demographics, and RE interventions), several previous reports have investigated the regulation of basal and postprandial MPS via secondary research methods [28–30]. Therefore, with the broad aim of providing an evidence summary for researchers and practitioners, the purpose of the present study was threefold: (1) consolidate the literature that has measured the MPS response to RE; (2) of these, characterise the magnitude and time course of the MPS response to RE; and (3) identify and discuss study-level covariates (e.g., participant characteristics, experimental factors, and RE parameters).

## 2. Methods

### 2.1. Literature Search Strategy

The search strategy was informed by the Preferred Reporting Items for Systematic Review (PRISMA) guidelines and a Population, Intervention, Comparison, Outcome, and Study type (PICOS) framework was used to determine the search strategy and study characteristics (SM: Table S1). Electronic database searches were performed through PubMed (Medline), Web of Science, Embase, Sport Discus, and Cochrane databases on 1 September 2021 and then updated on 3 July 2023. Title/Abstract/Keyword search terms used were as follows: (1) intervention (i.e., “resistance exercise,” “resistance training,” “strength exercise,” “weightlifting,” “knee extension exercise”); (2) outcome (i.e., “muscle protein synthesis,” “myofibrillar protein synthesis,” “fractional synthetic rate,” “fractional synthesis rate,” “mixed protein synthesis,” “protein synthetic rate”). Boolean operator “OR” was used between concept terms, and “AND” was used to combine constructs 1 and 2. Additionally, the reference lists of papers identified were checked for additional relevant papers, as well as reference lists of previous review papers related to this topic. Search information is reported in the Supplementary Material (SM: Appendix S1). The search was performed independently by two co-authors (AEL and UK), and potential conflicts between co-authors were resolved by consulting with a third co-author (RWD).

### 2.2. Eligibility Criteria

The eligibility criteria for this review were as follows: (1) published in peer-reviewed, English language journals; (2) healthy adult humans, nonobese; (3) RE performed, operationally defined as a single bout of exercise against external resistance applied to a targeted muscle group; (4) a validated and direct method of measuring mixed muscle and/or myofibrillar protein synthesis (e.g., precursor-product methods using labelled amino acids or D_2_O), and indirect estimates of MPS, such as measures of whole-body protein synthesis, net balance, and/or protein turnover, were not included; (5) fasted/postabsorptive state assessment of MPS (e.g., dietary intervention, ingestion of supplement, or standard meals during assessment period, Refs. [5, 9–11, 33–35] were excluded), ingestion of non-nutritive/noncaloric placebos were permitted, (e.g., water, noncaloric artificially flavoured water, and cellulose); (6) within-subject fasted/resting (i.e., basal) MPS comparator, obtained under the same experimental/physiological state/conditions in close temporal proximity, (e.g., pretest bilateral/unilateral or contralateral non-RE limb, Refs. [36–40] were excluded); (7) studies that utilised primary and/or adjuvant interventions in conjunction with RE were excluded (e.g., aerobic exercise, high-intensity interval training, blood flow restriction training, hypoxic training, pharmaceuticals, and ergogenic aids); and (8) data not published elsewhere (pseudo-replication), e.g., [41]. The only outcome of interest was the unstandardised effect size derived from basal and RE measurements of MPS. Studies that did not report MPS data were not included in the review.

### 2.3. Data Collection and Analysis

#### 2.3.1. Selection of Studies

References generated from the literature search were managed using the reference management software package EndNote™ (Thomson Reuters, v20). After compiling the initial EndNote™ library, duplicate references were removed using the “Find Duplicates” function, as well as manually screening for additional duplicate references that were not automatically removed. Titles and abstracts were then screened for eligibility, and studies deemed ineligible at this stage of the review were excluded. The full-text articles of the remaining studies were obtained for full-text screening and completed independently by two co-authors (AEL and UK) using the eligibility criteria outlined above. The level of agreement between the two co-authors was deemed as “very good” (Cohen's *κ* = 0.85) [42]. Any disagreements were individually examined and, if necessary, arbitrated by a third co-author (RWD).

#### 2.3.2. Data Extraction and Management

Participant characteristics, resistance exercise parameters, and study characteristics were extracted into a customised spreadsheet (Microsoft Excel™). Data extracted included: (1) participant characteristics (i.e., chronological age, sex, and RE training status); (2) RE loading parameters (i.e., number of sets, repetitions per set, intensity, rest interval, endpoint) and other RE parameters (i.e., contraction type and muscle group); and (3) study characteristics (i.e., lead author, year of publication, sample size, biopsied muscle, muscle protein type, resting control type, biopsy time, measurement times, resting MPS, RE MPS, funding source, and authors' conflict of interest). Three composite variables were calculated: (1) “volume” (i.e., the total number of repetitions = repetitions per set × number of sets); (2) “workload” (i.e., volume × intensity); and (3) “work-to-rest ratio” (W : R) (i.e., workload ÷ total rest period). Only data localised to the exercised muscle were extracted for analysis [43]. Where data are defined as “not reported” (NR), authors could not be contacted and/or did not respond to information requests. Discrepancies between co-authors (AEL and UK) were examined, and agreement was reached by consensus with a third co-author (RWD). Individual standard errors (SE) or confidence intervals (CI) were converted to standard deviation (SD) units prior to data entry [44]. Measurement times are reported relative to the immediate onset of RE cessation (i.e., 0 h). Single-point estimate (i.e., weighted arithmetic mean) was calculated for RE bout parameters that varied (e.g., different number of reps per set, number of sets per exercise, and rest period between sets), and load prescription for RE was converted to %1 RM via the Epley formula where necessary (e.g., 10 RM load∼75% 1 RM) [45–47].

#### 2.3.3. Risk of Bias

Due to the nature of the RE intervention (i.e., which cannot be adequately blinded) and the inclusion of mixed experimental designs (ipsilateral pretest and post-test, unilateral-contralateral, and subgroups), a quality assessment tool for before-after (pre-post) studies (no control group) was deemed most appropriate to quantitatively assess the risk of bias (ROB) [48, 49]. Questions 11 and 12 were omitted as they were not applicable to eligible studies. ROB was assessed by two co-authors (AEL and RWD). No studies were excluded due to ROB.

#### 2.3.4. Data Synthesis and Analysis

Unstandardised mean differences (MDs) and 95% CIs were calculated for each trial [50, 51]. A random-effects model was used to calculate a pooled weighted mean difference (WMD) [95% CI] [52–56]. Heterogeneity was assessed using Cochrane's Q chi-square statistic (*Q*) and *I*^2^ [53]. Funnel plot symmetry was visually inspected (inverse-error method), and Egger's regression test was used to numerically assess publication bias [57, 58]. Exploratory random-effects subgroup meta-analysis (discrete covariates), random-effects meta-regression, and subgroup meta-regression (continuous covariates) were performed to assess the effect of potential moderators [59]. To reduce pseudo-replication and nonindependence, a single-point estimate (weighted MD and SE) was calculated from studies that reported multiple contiguous measures of MPS [60–62]. Results are displayed as MD and/or WMD (%·h^−1^) where appropriate with 95% CIs. *Z*-tests were used to examine whether individual WMD was statistically significant. All analyses were performed on SPSS software (v.28).

## 3. Results

### 3.1. Literature Search

The results of the literature search and screening process for selecting studies that met the inclusion criteria for the review are reported in SM: Figure S1. The initial literature search generated 7,893 results, of which 2,357 were duplicates and subsequently removed. A total of 5,407 titles were also removed following the title and abstract screen upon identification of grounds for immediate exclusion (e.g., nonhuman studies, postprandial MPS assessment, and non-RE exercise intervention). Following the full-text screening, a further five studies were removed due to the inability to obtain the full text of a study. Of the remaining 124 potentially eligible studies, 21 were included in the review as they were deemed to meet all the eligibility criteria [8, 23, 24, 26, 60–74]. A total of 80 studies were excluded due to a nutrient feed/feeding protocol in all study groups. In addition, four potentially eligible studies had to be removed due to the unavailability of the data. As several articles contained >1 RE trial and/or repeated measurements of MPS, a total of 79 individual effects (*k*) were analysed.

### 3.2. Included Studies

Data from eligible studies are summarised in Table 1.

### 3.3. Risk of Bias and Sensitivity Analysis

ROB of eligible studies is summarised in SM: Table S2. All eligible studies were deemed to be of “good” quality. However, no study reported information pertaining to the justification of the sample size (criteria 5) and blinded outcome assessment (criteria 8). Eighteen studies received funding from national/institutional health research institutes in the United States, the United Kingdom, Canada, Japan, and Denmark. Five studies reported industry funding sources from health care or agri-food sectors [61, 69, 71, 72, 74]. Results from Egger's regression test and visual inspection of funnel plots revealed a symmetrical distribution (*P*=0.209) indicating no evidence of publication bias (SM: Figure S2). Removal of the statistical outliers and utilisation of different correlation coefficients of 0.1, 0.3, 0.7, and 0.9 did not alter the significance of the meta-analytical outputs (SM: Table S4).

### 3.4. Temporal Response to Resistance Exercise

**Figure 1:**
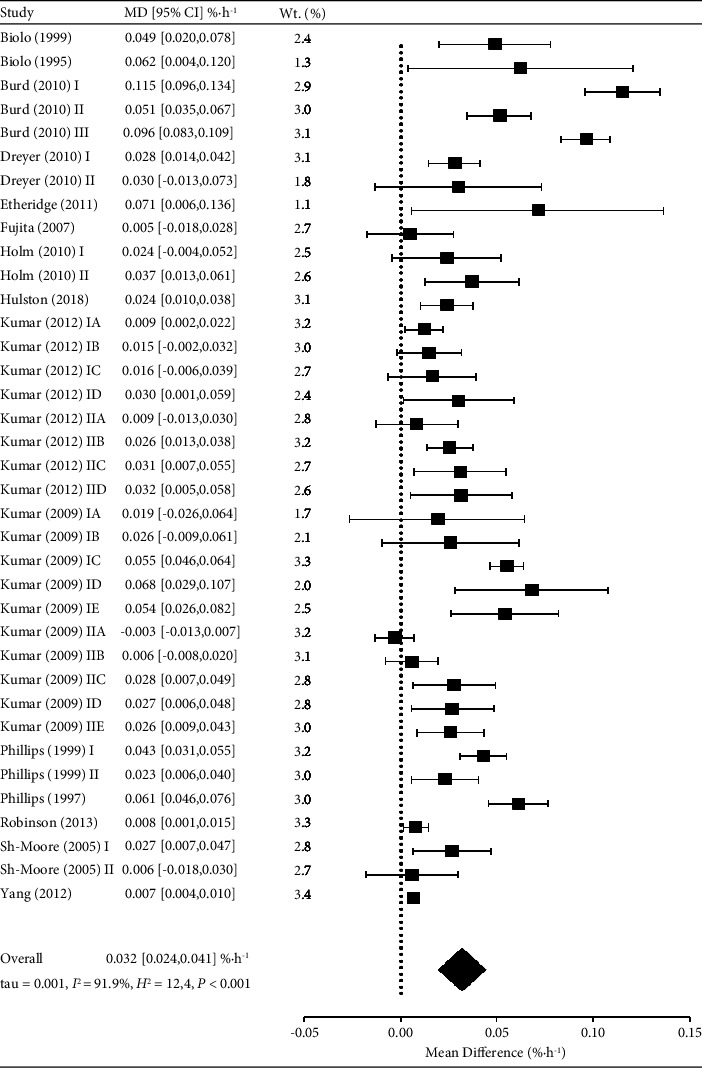
Forest plot of results from studies assessing the acute muscle protein synthesis (%·h^−1^) response to resistance exercise. Data are mean difference ±95% CI.

Fujita et al. [26] reported that MPS was attenuated below basal values during RE (MD: −0.019 [−0.033, −0.005] % h^−1^). Sheffield-Moore et al. [62] reported different age- and time-dependent MPS responses during RE, where older men had an acute increase in MPS peri-RE (MD: 0.044 [0.012, 0.076] % h^−1^), but younger men did not (MD: 0.000 [−0.008, 0.008] % h^−1^). Thereafter, the pooled analysis revealed robust increases in MPS were generally observed in the immediate period after RE up to 5.5 h (WMD: 0.032 [0.024, 0.041] % h^−1^, *P* < 0.001, *I*^2^ = 92%, *k* = 37), equivalent to 77 [66, 75] % increase above basal rates of 0.045 (0.013) % h^−1^ (Figure 1). Three studies partitioned the acute post-RE recovery period into 2 to 3 further discrete contiguous time periods [60, 62, 69]. However, there was no consistent pattern across trials, seemingly influenced by participant age and/or different RE loading parameters (i.e., intensity and workload). Five of the six studies that analysed the MPS response to RE in the later recovery period (i.e., after >6 h post-RE) reported sustained increases in MPS >12 h [68], >24 h [17, 23, 64, 73], and >48 h [17] post-RE.

### 3.5. Experimental Approach

All studies sampled muscle tissue from the VL, but Trappe et al. [72] reported an increase in MPS in m. soleus in young men after plantar flexion RE. Most eligible studies analysed either mixed muscle (43% of total participants) or myofibrillar (57% of total participants) protein synthesis, and two studies measured both [64, 68]. In terms of the resting control, one study used pooled bilateral data, and one study used both ipsilateral and contralateral controls. Eight studies used a unilateral RE with a contralateral control (30% of total participants), and 11 studies used a pre-exercise ipsilateral control (59% of total participants).

### 3.6. Participant Characteristics

#### 3.6.1. Age

Three eligible studies directly examined the effects of age on the MPS response to RE [61, 62, 69]. Of the 237 participants, 174 (73%) were categorised as younger (<35 y) and 63 (27%) as older (>50 y). Subgroup meta-analysis revealed that post-REVLMPS was elevated in both younger (WMD: 0.041 [0.030, 0.052] % h^−1^, *P* < 0.001, *I*^2^ = 88%, *k* = 25) and older (WMD: 0.015 [0.007, 0.022] % h^−1^, *P*=0.002, *I*^2^ = 76%, *k* = 12) adults. However, the magnitude of the increase was greater in the younger adults (93 [79, 108] % vs. 44 [37, 50] %, *Z* = 3.818, *P* < 0.001) (Figure 1).

#### 3.6.2. Sex

Twelve per cent of participants were women (*n* = 29) from one female-only study and four mixed-sex studies. Dreyer et al. [65] analysed the MPS response to RE between sexes, reporting MPS increases after RE with no difference between young men and women.

#### 3.6.3. Resistance Training Experience

Although most studies reported participants as “recreationally active,” only two studies defined participants as RE “trained” prior to enrolment (i.e., partaking in RE for at least 6 months 3 × week, ∼11% total participants) [64, 70]. Both studies reported that prior resistance training experience affected MPS with Phillips et al. [70] reporting an attenuated RE-induced increase in MPS in the trained participants compared to their untrained counterparts and Kim et al. [68] reporting a blunted response to RE (late-phase, mixed muscle not myofibrillar PS) in the trained state vs. untrained state.

### 3.7. Resistance Exercise Parameters

#### 3.7.1. Contraction Type

All but one study used “isoinertial” RE (i.e., moving a fixed mass object) with the exception of Etheridge et al. [24], which used an isometric knee extensor RE protocol. All but one study [72] used knee extensor RE (i.e., leg extensions and leg presses) either bilaterally (26% of total participants) or unilaterally (74% of total participants). Although no eligible study directly investigated the effect of unilateral vs. bilateral RE on MPS, exploratory subgroup meta-analysis showed that both bilateral (WMD: 0.032 [0.013, 0.050] % h^−1^, *P*=0.005, *I*^2^ = 72%, *k* = 8) and unilateral (WMD: 0.032 [0.022, 0.043] % h^−1^, *P* < 0.001, *I*^2^ = 94%, *k* = 29) RE stimulated MPS to a similar degree (*Z* = 0.098, *P*=0.922).

Eccentric-only RE was performed in four studies (16% of total participants) [17, 23, 70, 73]. These studies reported increases in MPS, which ranged from 0.016 to 0.058% h^−1^. One study used isometric RE [24] reporting a 0.071% h^−1^ increase in MPS. One study combined independent eccentric-only and concentric-only RE interventions into a single group for analysis [17], as they reported no difference in MPS response to RE between eccentric-only and concentric-only interventions, where intensity (i.e., 80% 1 RM) and all other RE variables were matched. The remaining sixteen studies (83% of total participants) all used “isotonic” muscle actions (i.e., sequential eccentric and concentric muscle contractions).

#### 3.7.2. Loading Parameters

The total number of sets per muscle group ranged from 3 to 12 with most studies prescribing 3 to 6 sets total (59% of total participants). Reps per set ranged from 3 to 36, with most studies prescribing an 8 to 14 rep range (61% of total participants). Training intensity ranged from 16 to 120% concentric 1 RM, with most studies prescribing 70 to 80% 1 RM (56% total participants). The rest period between sets ranged from 0.5 to 4 min with most studies opting for 2 to 3 minutes of rest (81% of total participants). A fixed workload requirement was prescribed by 20 studies (92% of total participants), whereas two studies used (maximal) effort end point at a fixed intensity (i.e., momentary failure defined as the inability to complete a repetition at a prescribed load) [64, 67]. Results from the random-effects univariate meta-regression showed no moderating effect for any of the analysed continuous covariates (i.e., number of repetitions per set, number of sets, RE intensity, rest period, volume, workload, or W : R) (*P* > 0.185) (SM: Table S3).

## 4. Discussion

**Figure 2:**
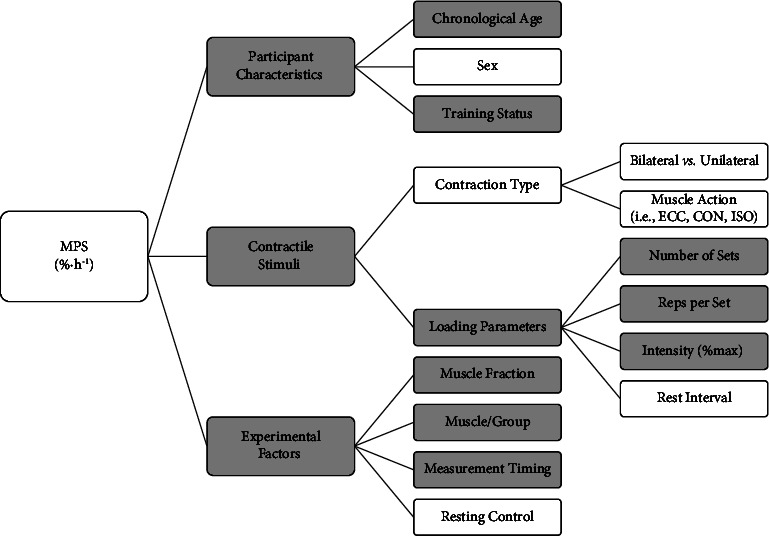
Overview of the covariates analysed and their putative effect on the acute muscle protein synthesis (MPS, % h^−1^) to resistance exercise, postabsorptive state. ECC, eccentric; CON, concentric; ISO, isometric muscle action. Grey shading indicates supporting evidence; no shading indicates an absence of, or limited, supporting evidence. N.b., absence of evidence is not evidence of absence; figure does not include composite, extraneous, or interaction between covariates; quality and quantity of supporting evidence varies between covariates.

This review aimed to synthesise and explore the MPS response to a single bout of RE in healthy adult humans. Pooled data collected from 79 controlled trials indicate that MPS is attenuated during RE, followed by an increase post-RE, which can be sustained past 24 h. Exploratory pooled subgroup meta-analysis showed that the magnitude of the RE-induced increase in MPS in healthy older adults is less than half that of their younger counterparts (93 [79, 108] % vs. 44 [37, 50] %). Moreover, we have little conclusive evidence, from pooled data analysis, to suggest any other demographic characteristic or RE variable-moderated MPS response to RE. However, results from individual studies have demonstrated that MPS response to RE varies across different muscle groups, between different muscle protein fractions, and is affected by participant training status and the RE loading parameters (i.e., intensity, workload, and effort). Therefore, to characterise the MPS response to RE, we have assimilated results from both the pooled data and individual study-level analysis for the discussion (Figure 2).

### 4.1. Time Course of the MPS Response to Resistance Exercise

There is consistent evidence here and elsewhere to suggest that MPS is attenuated during RE in the postabsorptive state [26, 76–78], which then returns to or above basal levels within 1 h post-RE [12, 26, 62, 78]. Pooled data from the present study demonstrates a robust increase in the rate of MPS thereafter (+77 [66, 75] %). However, there is substantial heterogeneity in the magnitude (0- to 2.7-fold increase) and time course (persisting for 3 to 48 h post-RE) of the increase in MPS. Pooled analysis of 17 controlled trials that measured the time course of the MPS response RE mostly reported peak values occurring in the first few hours post-RE [17, 60–62, 64, 69]. However, the time course thereafter was inconsistent with studies reporting either a return to baseline levels within 3 h [60–62, 69], or sustained increases past 3 h [60, 62, 69], 12 h [68], and >24 h [17, 23, 64, 72] post-RE. Five independent studies reported that the magnitude and time course were influenced by the RE loading parameters (i.e., intensity, volume, workload, and effort) and/or participant age [60–62, 64, 69]. Moreover, factors that affect the availability of the EAA preceding, during, or in between measurements (e.g., duration of the fasting period, dietary regimen trials, and EAA metabolism) were not consistently controlled or reported, and may therefore account for some of the variation, in the magnitude and duration of the MPS response to RE, both within and between trials [2, 60].

### 4.2. Resistance Exercise Training Variables

Unilateral exercise confers several benefits over bilateral exercise protocols from an experimental standpoint (e.g., reduced cost, time, and threats to internal validity) [79]. However, because of purported deficiencies of unilateral RE (e.g., bilateral deficit and/or lower postexercise circulating hormone concentrations), its efficacy has been questioned when compared to bilateral RE [79–81]. To our knowledge, no study has directly investigated the effect of unilateral vs. bilateral RE on MPS. However, a crude pooled analysis from the present study (i.e., not correcting for any other factors) shows that there was no difference between bilateral and unilateral RE, with both modes of RE stimulating MPS to a similar degree.

Most studies opted for RE that involved lifting and lowering submaximal fixed mass loads, which *de facto* splits the workload equally between concentric and eccentric phases of each repetition. Indeed, disparities between concentric and eccentric RE have been demonstrated across a broad range of muscular assessments (e.g., hypertrophy, strength, and remodelling) [82]. However, independent studies have reported no differences between concentric and eccentric RE on post-RE MPS when workload and (submaximal) intensity were matched, in both postabsorptive [17] and postprandial states [83]. Conversely, time-dependent differences have been reported between (supra)maximal eccentric RE and workload-matched concentric RE, where eccentric contractions evoked greater MPS post-RE [84]. Two independent studies included in this review directly analysed the MPS response following supramaximal eccentric RE (120% 1 RM). Cross-study evaluation of individual effects, against comparable isotonic/isometric RE studies (i.e., time, age, training status, workload), showed no further enhancement of MPS. Based on these findings, we have limited conclusive evidence here to suggest that any specific contraction type is superior at stimulating MPS. In addition, disparities are possibly influenced by other factors related to the RE loading parameters (e.g., intensity, workload, effort, and endpoint) rather than the type of muscle contraction *per se*.

The results from our exploratory pooled analysis indicate that the number of repetitions per set, number of sets, RE intensity, rest period, volume, workload, or W : R did not affect the post-RE MPS response. On the contrary, several individual studies have independently demonstrated that the manipulation of one or more RE variables can affect MPS. Four eligible studies included in this review investigated the effect of different workload-matched RE intensities on postexercise MPS [60, 61, 64, 69]. Indeed, Holm et al. [60] reported differences in the time course between low- (16% 1 RM) and high-intensity (70% 1 RM) RE. However, over the entire measurement period (0 to 5.5 h), there did not appear to be any difference between high and low loading intensities. Conversely, three studies reported greater MPS following high-intensity RE (i.e., 60 to 90% 1 RM) compared to workload-matched low-intensity RE (i.e., 30% to 40% 1 RM) [61, 64, 69]. Indeed, at the same intensity, there is evidence to suggest that MPS increases with workload (i.e., a higher number of reps per set and/or total number of sets) [61, 85]. However, the full dose response for volume has not been measured in humans, as it has for intensity [61]. To the authors' knowledge, only a murine model of RE (i.e., 10 electrically stimulated m. gastrocnemius contractions per set for 1, 3, 5, 10, and 20 sets) has quantified a dose-response for workload and MPS, revealing that MPS plateaued between 3 and 5 sets [86].

The conflicting results between workload, volume, and/or intensity-matched trials may be because when taken to the limit of “exercise tolerance” (i.e., task failure), the relationship between intensity and volume/workload is hyperbolic, not linear [87, 88]. Consequently, greater total volume/workloads can be attained at lower intensities prior to task failure (and presumably across all levels of effort). Although several studies reported using maximal workload RE protocols, stating that “task failure” occurred during RE, limited/inconsistent reporting/availability of data pertaining to the “proximity to task failure” meant it was not possible to evaluate its effect on MPS here [89]. However, the study of Burd et al. [64] demonstrated that differences in MPS in workload-matched low-intensity (30% 1 RM) and high-intensity RE (90% 1 RM) could be equalised by increasing training volume/workload output (∼50%) by matching the level of effort (i.e., maximal, performing RE to momentary failure).

### 4.3. Participant Characteristics

Results from our pooled analysis in addition to numerous independent trials clearly suggest that increases in MPS following RE were observed in both older and younger adults. However, age-related anabolic “resistance” or “blunting” (i.e., lower MPS response to RE) has been previously reported and discussed at length [12, 90, 91]. Three independent studies included in this review directly examined the effects of age on the MPS response to RE [61, 62, 69]. One comprehensive study reported lower MPS rates in older adults across a series of workload-matched RE protocols at different training intensities [61]. However, the other two studies reported conflicting findings. Sheffield-Moore et al. [62] reported time-dependent MPS response, with older adults having greater MPS during and just after RE but dropping below younger adults >1 hour post-RE; however, there were no differences over the entire 3-h post-RE measurement period. Kumar et al. [69] also reported that MPS was greater in younger men following workload-matched low-volume/low-intensity RE (3 sets, 40% 1 RM), but conversely not after low-volume/high-intensity RE (3 sets, 75% 1 RM), or high-volume RE (6 sets at any intensity). Taken together, these findings suggest that age-related “anabolic blunting” to RE is present, but can potentially be offset by manipulating RE loading parameters (i.e., increasing workload). Collectively, our exploratory pooled subgroup analysis, of 37 individual trials, revealed the magnitude increase in MPS in older adults was less than half that of their younger counterparts.

Pooled analysis of the effect of sex and training status on RE MPS could not be conducted due to incomplete reporting and limited availability of data. Only two of the five mixed-sex studies included in this review analysed the response between young men and women, neither study reported any difference between sexes following workload-matched RE [26, 65]. The absence of any sex difference, in young healthy adults, has also been reported in the postprandial state MPS response to RE [92].

Two independent studies included in this review directly investigated the effect of RE training experience on RE-induced MPS. Phillips et al. [70] reported lower rates of MPS in a cohort trained (i.e., ≥5 y RE experience) compared to untrained (i.e., no RE training experience whatsoever) participants, following a bout of supramaximal eccentric RE performed at the same relative intensity (i.e., greater absolute workload performed by the trained group due to a higher 1RM). Additionally, Kim et al. [68] longitudinally assessed the MPS response to RE (∼12 h post-RE) before and after 8 weeks of RE training, reporting a blunted MPS response to RE in the trained state. However, this result was not consistent across different muscle protein fractions, as RE training attenuated the mixed muscle but not myofibrillar protein synthesis response to RE [68]. Tang et al. [75] reported that the attenuated trained-state mixed MPS response to RE was caused by shortening the duration for which MPS was elevated, whereas the postprandial MPS response to RE was in fact greater in the trained versus the untrained state.

### 4.4. Other Factors

Results from the present study are principally limited to a single muscle/muscle group (i.e., knee extensors and the m. vastus lateralis). Although, to the authors' knowledge, no direct comparative assessment has ever been made between different muscle groups, the MPS response to exercise has been independently characterised in other muscles/muscle groups (e.g., soleus [93], biceps brachii [4, 6, 7], and deltoid [94]). Indeed, Trappe et al. [72] previously reported that the increase in MPS in the m. soleus in young men following plantar flexion RE was lower compared to data from several independent studies that independently measured the MPS response to RE in the VL. The observations made by Trappe et al. [72] are corroborated here (SOL: 0.018% h^−1^ vs. VL: 0.032% h^−1^) with our larger dataset concerning the VL MPS response to RE.

Generally, it is considered that the myofibrillar and mixed muscle MPS are interchangeable as the myofibrillar fraction accounts for ∼65% of muscle proteins [95]. Although most of the eligible studies included in this review, assessed either mixed or myofibrillar fractions, it is important to note that the synthetic response across the different muscle protein fractions is not uniform. Studies by Burd et al. [64] and Kim et al. [68] revealed differences in mixed muscle and myofibrillar MPS responses to RE in the late recovery period (>12 h), with mixed MPS generally being more responsive to RE than the myofibrillar fraction—but not under all conditions (i.e., no apparent increase in MPS immediately post-RE after a period of RE training, or following low-load exhaustive RE) [64, 68]. Similar observations have also been made in the postprandial state, which is presumably due to disparate synthetic responses to RE of the myofibrillar and nonmyofibrillar muscle fractions (e.g., sarcoplasmic, stromal, and/or mitochondrial) [13, 33, 64, 96].

### 4.5. Study Quality and Risk of Bias

All but one study [63] was assigned as nonrandomised control trials, as basal MPS was conventionally measured immediately prior to RE. That said, there are several reasons why randomisation is not preferred in this instance, because of unidirectional order effects (i.e., as a result of RE) necessitating an unknown and likely prolonged “wash-out” period between measurements [60, 68]. Furthermore, regulation of MPS, to our knowledge, is not prone to expectation effects and volitional exercise is *de facto* impossible to adequately blind. However, considering the ostensibly equivocal MPS response between bilateral and unilateral RE, the use of a unilateral RE model (i.e., two limbs randomised to one of two treatments) can potentially overcome threats to quality/ROB related to nonrandomisation (i.e., time and/or treatment order effects). Aside from issues related to blinding and randomisation, generally low ROB was observed across all studies included in this review. Nevertheless, we identified three reporting issues that were consistent across the majority of eligible studies: (1) clear justification of the sample size to provide confidence in the findings; (2) clear declaration that the assessors were blind to the treatment condition during analysis; (3) clear description in the reporting of the basic prognostic RE parameters that influence MPS (e.g., contraction velocity/time under tension [97], duty cycle [98], and effort/proximity to task failure [64]).

### 4.6. Limitations

In addition to the general limitations that can be applied to the statistical and methodological approaches that have been used here, which are discussed in detail elsewhere [12, 52, 99], there are also several specific limitations we wish to acknowledge. First, results from this study are defined, and thus restricted to, eligibility criteria and eligible study data (i.e., healthy adult humans, resistance exercise only, postabsorptive state, and principally the acute post-RE VL MPS response to knee extensor RE). The exclusion of postprandial MPS data was conducted to deduce the *singular* effect of RE and facilitate exploratory cross-study analysis. We did not deem it viable or valid, to control or correct for variation in the different feeding protocols (i.e., dose, frequency, timing, and type) between studies that would confound the RE effect. Nevertheless, where relevant, we have drawn evidence from multitrial studies that used a standardised feeding protocol (i.e., between groups/trials), to support findings made in the present review [75, 83, 92]. That said, it should be noted that there are fundamental differences in postabsorptive and postprandial MPS responses to RE that may alter both the magnitude and time course of it. For example, the reported attenuation of the postabsorptive rate of MPS during RE can be eliminated, and the subsequent post-RE time course altered, with specific feeding strategies [100]. Second, where appropriate point estimates were employed for some variables to minimise bias (i.e., nonindependence) during cross-study and/or pooled-study evaluation. However, despite best efforts, it should be noted that pooled effects may be biased towards multitrial studies.

Lastly, we would like to stress that results from this review are limited to acute experimental trials conducted under controlled laboratory postabsorptive conditions, characterising the acute MPS response to a *single bout* of RE. The findings from this review, investigating the acute MPS response to RE, should not be confused/conflated with the longer-term muscular responses measured in “free-living” or “real-world” settings in response to RE *training* interventions (i.e., repeated bouts of RE). These studies naturally incorporate other (extraneous) factors in addition to the RE *per se*, which do not factor in acute lab-based assessments of MPS (e.g., dietary intake and feeding pattern, nonexercise physical in/activity, sleep pattern, stress, medications, hormonal/diurnal variation, training frequency and duration, recovery, compliance, progression, and periodisation of RE training bout-to-bout) [11, 35, 101]. Although detailed discussion regarding this matter is beyond the scope of the current review (see Refs. [3, 31, 32, 102, 103]), the acute synthetic response to RE can be used to inform and gain mechanistic insight into the dynamic remodelling, repair, regenerative, and/or growth responses to RE [9, 27, 31, 60, 68, 75, 97, 104] and its potential moderators (e.g., ageing, training, disease, nutritional status, micro/environment, and contractile stimuli) [2, 5, 11, 12, 20–26, 36, 40, 100], which cannot be detected/deduced from crude/static muscle measurements (e.g., lean mass, cross-sectional area, muscle thickness/volume from DXA, ultrasound, MRI, or CT scans).

## 5. Conclusion

The evidence consolidated in this exploratory analysis suggests that there is a phasic MPS response to RE in healthy humans, measured under postabsorptive conditions. An attenuation of MPS occurs during RE, which is followed by a measurable increase above basal levels that can be sustained past 24 h post-RE. However, there is substantial heterogeneity in the magnitude and time course of the MPS response reported between trials. Known sources of variation include participants' age, RE training experience, and RE loading parameters (e.g., intensity, volume, and effort) (Figure 2). Nevertheless, most eligible studies included in this review adopted a “standard” model of RE, which consisted of 3 to 6 sets of 8 to 14 repetitions of moving a fixed mass object (70 to 80% 1 RM) at a high to maximal level of effort with 2- to 3-min rest between working sets. Results from the present investigation tentatively suggest that departure from this “benchmark” provides any further enhancement of MPS in humans. However, similar rates of MPS can be attained different RE intensities or contraction types provided the workload and level of effort are comparable. The MPS response following RE appears to be lower in older adults and trained adults but can be counteracted, to varying degrees of success, by higher work outputs (e.g., more intense and/or greater volume of RE). Moreover, there is limited conclusive evidence to suggest the RE-induced increase in MPS differs between young men and women, or between different types of muscle contraction. We hope this information can be used by scientists and practitioners to inform future RE research and practices focusing on the acute MPS response to RE and its corollaries.

## 6. Perspective

MPS is the principal driving force underpinning the adaptive response to RE [2]. Thus, acute measures of MPS can be used to inform and gain mechanistic insight into the dynamic remodelling, repair, regenerative, and/or growth responses to RE and its potential moderating factors (e.g., ageing, training, disease, nutritional status, micro/environment, and contractile stimuli) [3, 12]. Here, we provide some valuable information regarding the time course, magnitude, and pattern of the MPS response to RE and its potential covariates (Figure 2). Factors that researchers and practitioners should consider are the participants'/clients' chronological age, prior RE training experience, the time frame of the recovery period, and the loading parameters of the RE itself, whereas sex and the type of muscle contraction appear less influential. Researchers and practitioners may wish to use the information provided in this review to improve the practice, quality, and efficiency of their work.

## Figures and Tables

**Table 1:** Main characteristics of eligible studies.

Study	Participants	Muscle sample and control	Contraction	Sets × reps × intensity (%) × rest (min)	MD (CI) % h^−1^ tracer	Meta-analysed
Biolo et al. [63]	5 men 29 years Untrained	VL Mixed PRE	Isotonic Bilateral Knee extensions	14^‡^ × 9^‡^ × 72^‡†^ × 2.5^‡^	0–3 h 0.049 [0.020, 0.078] Tracer: [^13^C_6_]Phe	Yes

Biolo et al. [8]	5 men 24 years Untrained	VL Mixed PRE	Isotonic Bilateral Knee extensions	14^‡^ × 9^‡^ × 72^‡†^ × 2.5^‡^	0–3 h 0.062 [0.004, 0.120] Tracer: [^13^C_6_]Phe	Yes

Burd [23]	8 men 23 years Untrained	VL Mixed PRE	Eccentric Unilateral Knee extensions	10 × 10 × 120^‡^ × 1	24–27 h 0.016 [0.007, 0.025] Tracer: [^2^H_5_]Phe	No

Burd et al. [64]	15 men 21 years Trained	VL Mixed + Myo PRE	Isotonic Unilateral Knee extensions	I: 4 × 5^‡^ × 90 × 3 II: 4 × 14^‡^ × 30 × 3 III: 4 × 24^‡^ × 30 × 3	0–4 h Mixed I: 0.115 [0.096, 0.134] II: 0.051 [0.035, 0.067] III: 0.096 [0.083, 0.109] Myo I: 0.063 [0.045, 0.081] II: 0.022 [0.011, 0.033] III: 0.071 [0.062, 0.083] 24–28 h Mixed I: 0.032 [0.015, 0.049] II: 0.030 [0.019, 0.041] III: 0.053 [0.042, 0.064] Myo I: 0.026 [0.017, 0.035] II: 0.007 [−0.002, 0.016] III: 0.051 [0.042, 0.060] Tracer: [^13^C_6_]Phe	Yes: 0–4 h mixed

Dreyer et al. [65]	Untrained I: 9 men 27 years II: 8 women 26 years	VL Mixed PRE	Isotonic Bilateral Knee extensions	I: 10 × 10 × 67 × 3 II: 10 × 10 × 66 × 3	0–2 h I: 0.028 [0.014, 0.042] II: 0.030 [−0.013, 0.073] Tracer: [^2^H_5_]Phe	Yes

Etheridge et al. [24]	7 men 21 years Untrained	VL Myo CL	Isometric Unilateral Knee extensions	6 × 8 × 70 × 2	0–2.5 h 0.071 [0.006, 0.136] Tracer: [^13^C_2_]Leu	Yes

Fujita et al. [26]	7 men + 4 women 27 years Untrained	VL Mixed PRE	Isotonic Bilateral Knee extensions	10 × 10 × 70 × 3	During RE −0.019 [−0.033, −0.005] Tracer: [^2^H_5_]Phe	No

Fujita et al. [26]	6 men 32 years Untrained	VL Myo PRE	Isotonic Bilateral Knee extensions	4 × 19^‡^ × 20 × 0.5	During RE–3 h 0.005 [−0.018, 0.028] Tracer: [^13^C_6_]Phe	Yes

Hansen et al. [66]	10 women 60 years Untrained	VL Myo CL	Isotonic Unilateral Knee extensions	10 × 10 × 75^†^ × 3	24–26 h −0.008 [−0.019, 0.003] Tracer: [^13^C]Pro	No

Holm et al. [60]	10 men 26 years Untrained	VL Myo PRE	Isotonic Unilateral Knee extensions	I: 10 × 36 × 16 × 0.5 II: 10 × 8 × 70 × 3	0.5–3 h I: 0.035 [0.008, 0.062] II: 0.009 [−0.010, 0.028] 3–5.5 h I: 0.013 [−0.017, 0.043] II: 0.065 [0.035, 0.095] Tracer: [^13^C]Leu	Yes: 0.5–3 h

Hulston et al. [67]	8 men + 1 woman 27 years Untrained	VL Mixed CL	Isotonic Unilateral Knee extensions	4 × 9^‡^ × 70 × 3	0–4 h 0.024 [0.010, 0.038] Tracer: [^13^C_6_]Phe	Yes

Kim et al. [68]	8 men 25 years I: Untrained II: Trained	VL Mixed + Myo PRE	Isotonic Unilateral Knee extensions	8 × 10 × 80 × 2	12–16 h Mixed I: 0.053 [0.031, 0.075] II: 0.011 [−0.003, 0.025] Myo I: 0.012 [0.009, 0.015] II: 0.012 [0.006, 0.018] Tracer: [^13^C_6_]Phe	No

Kumar et al. [69]	24 men Untrained I: 24 years II: 70 years	VL Myo PRE	Isotonic Unilateral Knee extensions	A: 3 × 14 × 40 × 3 B: 6 × 14 × 40 × 3 C: 3 × 8 × 75 × 3 D: 6 × 8 × 75 × 3	0-1 h IA: 0.002 [−0.011, 0.015] IB: 0.021 [−0.005, 0.047] IC: 0.002 [−0.023, 0.027] ID: 0.032 [−0.014, 0.078] IIA: 0.013 [−0.002, 0.028] IIB: 0.042 [0.024, 0.060] IIC: −0.016 [−0.031, −0.001] IID: 0.030 [0.015, 0.045] 1-2 h IA: 0.027 [0.018, 0.036] IB: 0.005 [−0.018, 0.028] IC: 0.059 [0.015, 0.103] ID: 0.082 [0.045, 0.119] IIA: 0.005 [−0.010, 0.020] IIB: 0.052 [0.022, 0.082] IIC: 0.032 [0.009, 0.055] IID: 0.054 [0.004, 0.104] 2–4 h IA: 0.010 [0.001, 0.019] IB: 0.017 [0.008, 0.026] IC: 0.002 [−0.009, 0.013] ID: 0.003 [−0.012, 0.018] IIA: 0.008 [−0.020, 0.036] IIB: 0.015 [−0.008, 0.038] IIC: 0.029 [0.020, 0.038] IID: 0.021 [0.001, 0.041] Tracer: [^13^C_2_]Leu	Yes: 0–4 h

Kumar et al. [61]	Untrained I: 25 men 24 years II: 25 men 70 years	VL Myo PRE	Isotonic Unilateral Knee extensions	A: 3 × 27 × 20 × 2 B: 3 × 14 × 40 × 2 C: 3 × 9 × 60 × 2 D: 3 × 8 × 75 × 2 E: 6 × 3 × 90 × 2	1-2 h IA: 0.019 [−0.026, 0.064] IB: 0.026 [−0.009, 0.061] IC: 0.055 [0.046, 0.064] ID: 0.068 [0.029, 0.107] IE: 0.054 [0.026, 0.082] IIA: −0.003 [−0.013, 0.007] IIB: 0.006 [−0.008, 0.020] IIC: 0.028 [0.007, 0.049] IID: 0.027 [0.006, 0.048] IIE: 0.026 [0.009, 0.043] Tracer: [^13^C_2_]Leu	Yes

Phillips (1999) [70]	6 men + 6 women I: 25 years, untrained II: 26 years, trained	VL Mixed CL	Eccentric Unilateral Knee extensions	8 × 10 × 120 × 3	0–4 h I: 0.043 [0.031, 0.055] II: 0.023 [0.006, 0.040] Tracer: [^2^H_5_]Phe	Yes

Phillips et al. [17]	4 men + 4 women 23 years Untrained	VL Mixed CL	Eccentric + Concentric^*∗*^ Bilateral Knee extensions	8 × 8 × 80 × 3	0–3 h 0.061 [0.046, 0.076] 24–27 h 0.036 [0.021, 0.051] 48–51 h 0.019 [0.006, 0.032] Tracer: [^2^H_5_]Phe	Yes: 0–3 h

Robinson et al. [71]	7 men 59 years Untrained	VL Myo CL	Isotonic Unilateral Knee extensions	3 × 8 × 75^†^ × 2	0–4 h 0.008 [0.001, 0.015] Tracer: [^13^C_6_]Phe	Yes

Sheffield-Moore et al. [62]	12 men Untrained I. 29 years II. 67 years	VL Mixed PRE	Isotonic Bilateral Knee extensions	6 × 8 × 80 × NR	During RE–0.2 h I. 0.000 [−0.008, 0.008] II. 0.044 [0.012, 0.076] 0.2–1 h I. 0.019 [0.007, 0.031] II. 0.013 [−0.010, 0.036] 1–3 h I. 0.030 [0.007, 0.053] II. 0.003 [−0.021, 0.027] Tracer: [^2^H_5_]Phe	Yes: 0.2–3 h

Trappe et al. [72]	8 men 27 years Untrained	SOL Mixed CL	Isotonic Unilateral Plantar flexions	12 × 15 × 70 × 2^‡^	0–3 h 0.018 [−0.004, 0.040] Tracer: [^2^H_5_]Phe	No

Trappe et al. [73]	6 men 25 years Untrained	VL Mixed PRE	Eccentric Unilateral Knee extensions	12^‡^ × 10 × 120 × 1	26–29 h 0.058 [0.046, 0.070] Tracer: [^2^H_5_]Phe	No

Yang et al. [74]	10 men 71 years Untrained	VL Myo PRE	Isotonic Unilateral Knee extensions	3 × NR × 75^†^ × 2	0–4 h 0.007 [0.004, 0.010] Tracer: [^13^C_6_]Phe	Yes

Data are mean (SD); MD = mean difference; CI = 95% confidence interval; VL = m. Vastus lateralis; SOL = m. soleus; Mixed = mixed muscle; Myo = myofibrillar; Intensity, % concentric 1 RM; ^‡^varied, data reported are the bout average; ^†^estimated via Epley formula; NR = not reported; PRE = pre-exercise control; CL = contralateral control; Phe = phenylalanine; Leu = leucine; Pro = proline.

## Data Availability

The data that support the findings of this study are available in Table 1, Figure 1, and the Supplementary Material of this article.

## Supplementary Materials

Supplementary Materials Figure S1: PRISMA flowchart. Appendix S1: Database search strategies. Table S1: PICOS inclusion criteria. Table S2: Risk of Bias Assessment. Table S3: Meta-regression output. Table S4: Sensitivity analysis. Figure S2: Funnel Plot.
