# Supplementary material for: Characterisation of the Muscle Protein Synthetic Response to Resistance Exercise in Healthy Adults: A Systematic Review and Exploratory Meta-Analysis
Source: Transl Sports Med. 2024 Apr 30;2024:3184356. doi: 10.1155/2024/3184356 (PMC11074832; doi:10.1155/2024/3184356)
Supplement: Supplementary Materials — Figure S1: PRISMA flowchart. Appendix S1: Database search strategies. Table S1: PICOS inclusion criteria. Table S2: Risk of Bias Assessment. Table S3: Meta-regression output. Table S4: Sensitivity analysis. Figure S2: Funnel Plot. [file 3184356.f1.docx]

**Supplementary Materials**

**Article Title**

Characterisation of the muscle protein synthetic response to resistance exercise in healthy adults: a systematic review and exploratory meta-analysis

**Author List**

Robert W. Davies^1^, Arthur E. Lynch^2^, Uttam Kumar^2^ & Philip M. Jakeman^2,3^

**Affiliations**

1. Chester Medical School, University of Chester, Shrewsbury, United Kingdom
2. Department of Physical Education and Sport Sciences, University of Limerick, Limerick, Ireland.
3. Health Research Institute, University of Limerick, Limerick, Ireland.

**Corresponding author**

Robert W. Davies; [r.davies@chester.ac.uk](mailto:r.davies@chester.ac.uk)

Records removed *before screening*:

Duplicate records removed

(n = 2357)

Records removed for other reasons (n = 0)

Records identified (n = 7893):

Cochrane (n = 694)

PubMed (n = 1903)

EMBASE (n = 2223)

SPORTDiscus (n = 1000)

Web of Science (n = 2073)

**Identification**

Records screened

(n = 5536)

Records excluded

(n = 5407)

Reports sought for retrieval

(Post title & abstract screen)

(n = 129)

Reports not retrieved

(n = 5)

**Screening**

Reports assessed for eligibility

(Post full text screen)

(n = 124)

Reports excluded

(n = 103)

- nutrient intervention (n = 80)
- unable to obtain data (n = 4)
- no control (n = 4)
- other (n = 15)

Studies included in review

(n = 21)

**Included**

**Fig. S1** – PRISMA flowchart documenting the different phases of the systematic review process

**Appendix S1**: Database search strategies

PubMed

Searched: 01/09/2021 updated 03/07/2023

“resistance exercise[Title] **OR** strength exercise[Title] **OR** weightlifting[Title] **OR** knee extension exercise[Title] **OR** elbow flexion exercise[Title]”

“**AND** muscle protein synthesis[Title/Abstract] **OR** fractional synthetic rate[Title/Abstract] **OR** fractional synthesis rate[Title/Abstract] **OR** protein synthetic rate[Title/Abstract] **OR** mixed protein synthesis[Title/Abstract]”

Embase

Searched: 01/09/2021 updated 03/07/2023

**1.** “resistance exercise” **OR** “resistance exercise training” **OR** "weightlifting" **OR** "strength exercise".

**2**. “muscle protein synthesis” **OR** "myofibrillar protein synthesis" **OR** "mixed protein synthesis" **OR** "fractional synthe* rate”.

**3.** (“Humans” **OR** “man” **OR** “woman”)

**4.** 1 and 2 and 3.

Cochrane

"resistance exercise"[Title/Abstract/Keyword] **OR** "resistance exercise training"[Title/Abstract/Keyword] **AND** muscle protein synthesis[Title/Abstract/Keyword] **OR** myofibrillar protein synthesis[Title/Abstract/Keyword] **AND** human[Title Abstract Keyword] - in Cochrane Protocols, Trials (Word variations searched)

Web of Science

Searched: 01/09/2021 updated 03/07/2023

“resistance exercise”[All fields] **AND** “muscle protein synthesis[All fields]”

Sport Discus (EBSCO)

Searched: 01/09/2021 updated 03/07/2023

“resistance exercise” **OR** “resistance exercise training” **OR** "weightlifting" **OR** "strength exercise") **AND**

“muscle protein synthesis” **OR** "myofibrillar protein synthesis" **OR** "mixed protein synthesis" **OR** "fractional synthe* rate") **AND**

“Humans” **OR** “man” **OR** “woman”

| Parameter description |
| --- |
| Population: healthy human adults ≥ 18 years of age |
| Intervention: resistance exercise and/or resistance exercise + non-nutritive placebo |
| Comparison: postabsorptive resting *vs.* resistance exercise muscle protein synthesis |
| Outcome: postabsorptive fractional synthetic rate of muscle protein synthesis (%·h^-1^) |
| Study Type: Within-subject repeated measures design |

**Table S1:** PICOS (Population, Intervention, Comparison, Outcome, Study Design) criteria for inclusion of studies

**Table S2**. Risk of Bias Assessment

| **Study** | **C1** | **C2** | **C3** | **C4** | **C5** | **C6** | **C7** | **C8** | **C9** | **C10** |
| --- | --- | --- | --- | --- | --- | --- | --- | --- | --- | --- |
| Biolo (1999) | Y | Y | Y | Y | NR | Y | Y | NR | Y | Y |
| Biolo (1995) | Y | Y | Y | Y | NR | Y | Y | NR | Y | Y |
| Burd (2010) | Y | Y | Y | Y | NR | Y | Y | NR | Y | Y |
| Burd (2010) | Y | Y | Y | Y | NR | Y | Y | NR | Y | Y |
| Dreyer (2010) | Y | Y | Y | Y | NR | Y | Y | NR | Y | Y |
| Etheridge (2011) | Y | Y | Y | Y | NR | Y | Y | NR | Y | Y |
| Fujita (2009) | Y | Y | Y | Y | NR | Y | Y | NR | Y | Y |
| Fujita (2007) | Y | Y | Y | Y | NR | Y | Y | NR | Y | Y |
| Hansen (2012) | Y | Y | Y | Y | NR | Y | Y | NR | Y | Y |
| Holm (2010) | Y | Y | Y | Y | NR | Y | Y | NR | Y | Y |
| Hulston (2018) | Y | Y | Y | Y | NR | Y | Y | NR | Y | Y |
| Kim (2005) | Y | Y | Y | Y | NR | Y | Y | NR | Y | Y |
| Kumar (2012) | Y | Y | Y | Y | NR | Y | Y | NR | Y | Y |
| Kumar (2009) | Y | Y | Y | Y | NR | Y | Y | NR | Y | Y |
| Phillips (1999) | Y | Y | Y | Y | NR | Y | Y | NR | Y | Y |
| Phillips (1997) | Y | Y | Y | Y | NR | Y | Y | NR | Y | Y |
| Robinson (2013) | Y | Y | Y | Y | NR | Y | Y | NR | Y | Y |
| Sheffield-Moore (2005) | Y | Y | Y | Y | NR | Y | Y | NR | Y | Y |
| Trappe (2004) | Y | Y | Y | Y | NR | Y | Y | NR | Y | Y |
| Trappe (2002) | Y | Y | Y | Y | NR | Y | Y | NR | Y | Y |
| Yang (2012) | Y | Y | Y | Y | NR | Y | Y | NR | Y | Y |

Y = yes; N = no; NR = not reported; C1 = Was the study question or objective clearly stated?; C2 = Were eligibility/selection criteria for the study population prespecified and clearly described?; C3 = Were the participants in the study representative of those who would be eligible for the test/service/intervention in the general or clinical population of interest?; C4 = Were all eligible participants that met the prespecified entry criteria enrolled?; C5 = Was the sample size sufficiently large to provide confidence in the findings?; C6 = Was the intervention clearly described and delivered consistently across the study population?; C7 = Were the outcome measures prespecified, clearly defined, valid, reliable, and assessed consistently across all study participants?; C8 = Were the people assessing the outcomes blinded to the participants' exposures/interventions?; C9 = Was the loss to follow-up after baseline 20% or less? Were those lost to follow-up accounted for in the analysis? C10 = Did the statistical methods examine changes in outcome measures from before to after the intervention? Were statistical tests done that provided p values for the pre-to-post changes?

**Table S3.** Results from the meta-regression analysis for the fractional synthetic rate of muscle protein synthesis response to resistance exercise.

| Covariate | Coefficient | SE | P-value | 95% CI | R^2^ (%) |
| --- | --- | --- | --- | --- | --- |
| Sets | 0.002 | 0.002 | 0.288 | -0.001 to 0.005 | 0.5 |
| Reps | -0.001 | 0.006 | 0.268 | -0.002 to 0.000 | 0.9 |
| Intensity | 0.022 | 0.016 | 0.185 | -0.011 to 0.055 | 2.4 |
| Rest | 0.007 | 0.006 | 0.281 | -0.006 to 0.019 | 0.5 |
| Volume | 0.000 | 0.000 | 0.833 | 0.000 to 0.000 | 0.0 |
| Workload | 0.000 | 0.002 | 0.650 | 0.000 to 0.000 | 0.0 |
| W:R | -0.003 | 0.003 | 0.317 | -0.008 to 0.003 | 0.1 |

W:R = work to rest ratio; SE = standard error; CI = confidence interval

| Analysis | 0.1 | 0.3 | 0.5 | 0.7 | 0.9 | Trimmed |
| --- | --- | --- | --- | --- | --- | --- |
| 3.4 | < 0.001 | < 0.001 | < 0.001 | < 0.001 | < 0.001 | < 0.001 |
| 3.6.1 | < 0.001 | < 0.001 | < 0.001 | < 0.001 | < 0.001 | < 0.001 |
|  | < 0.001 | < 0.001 | 0.002 | < 0.001 | < 0.001 | 0.002 |
|  | < 0.001 | < 0.001 | < 0.001 | < 0.001 | <0.001 | < 0.001 |
| 3.7.1 | 0.004 | 0.004 | 0.005 | 0.005 | 0.004 | 0.005 |
|  | < 0.001 | < 0.001 | < 0.001 | < 0.001 | < 0.001 | < 0.001 |
|  | 0.629 | 0.636 | 0.922 | 0.739 | 0.745 | 0.732 |
| 3.7.2 | 0.183 | 0.162 | 0.185 | 0.134 | 0.128 | 0.363 |

**Table S4.** Sensitivity analysis

Column 1, analysis sub-section reference; row 1, columns 2-6, within-subject correlation (r); column 7, trimmed data set with statistical outliers removed. Data are p-values (α = 0.05).

**
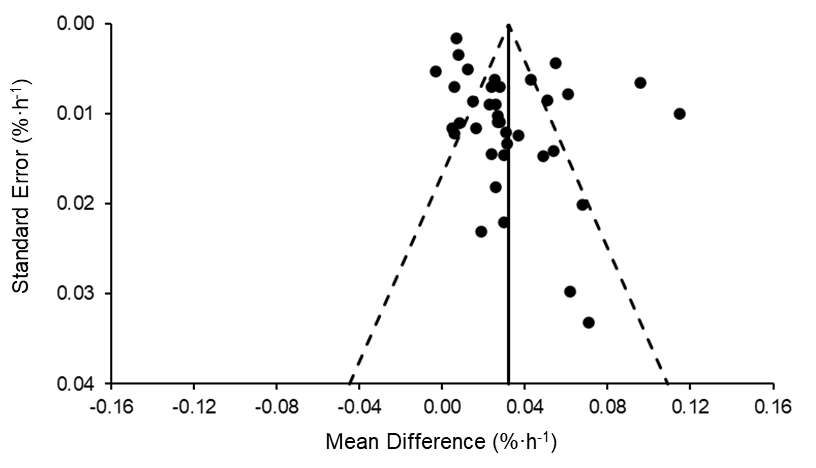
**

**Fig S2.** Funnel plot of inverse standard error against mean difference. Each circle represents an individual effect. Solid line is the weighted mean difference and triangular 95% CI (dashed line).

**References**

1. Biolo G, Maggi SP, Williams BD, et al. Increased rates of muscle protein turnover and amino acid transport after resistance exercise in humans. Am J Physiol. 1995;268:E514–20.
2. Biolo G, Williams BD, Fleming RY, Wolfe RR. Insulin action on muscle protein kinetics and amino acid transport during recovery after resistance exercise. Diabetes. 1999;48(5):949-57.
3. Burd NA, Dickinson JM, Lemoine JK, Carroll CC, Sullivan BE, Haus JM, et al. Effect of a cyclooxygenase-2 inhibitor on postexercise muscle protein synthesis in humans. Am J Physiol Endocrinol Metab. 2010;298(2):E354‐61.
4. Burd NA, West DWD, Staples AW, Atherton PJ, Baker JM, Moore DR, et al. Low-Load High Volume Resistance Exercise Stimulates Muscle Protein Synthesis More Than High-Load Low Volume Resistance Exercise in Young Men. PLoS ONE. 2010;5(8):e12033. doi: 10.1371/journal.pone.0012033.
5. Dreyer HC, Fujita S, Glynn EL, Drummond MJ, Volpi E, Rasmussen BB. Resistance exercise increases leg muscle protein synthesis and mTOR signalling independent of sex. Acta Physiol. 2010;199(1):71-81.
6. Etheridge T, Atherton PJ, Wilkinson D, Selby A, Rankin D, Webborn N, et al. Effects of hypoxia on muscle protein synthesis and anabolic signaling at rest and in response to acute resistance exercise. Am J Physiol-Endocrinol Metab. 2011;301(4):E697-E702.
7. Fujita S, Dreyer HC, Drummond MJ, Glynn EL, Volpi E, Rasmussen BB. Essential amino acid and carbohydrate ingestion before resistance exercise does not enhance postexercise muscle protein synthesis. J Appl Physiol. 2009;106(5):1730-9.
8. Fujita S, Abe T, Drummond MJ, Cadenas JG, Dreyer HC, Sato Y, et al. Blood flow restriction during low-intensity resistance exercise increases S6K1 phosphorylation and muscle protein synthesis. J Appl Physiol. 2007;103(3):903-10.
9. Hansen M, Skovgaard D, Reitelseder S, Holm L, Langbjerg H, Kjaer M. Effects of Estrogen Replacement and Lower Androgen Status on Skeletal Muscle Collagen and Myofibrillar Protein Synthesis in Postmenopausal Women. J Gerontol Biol Sci Med Sci. 2012;67(10):1005-13.
10. Holm L, van Hall G, Rose AJ, Miller BF, Doessing S, Richter EA, et al. Contraction intensity and feeding affect collagen and myofibrillar protein synthesis rates differently in human skeletal muscle. Am J Physiol-Endocrinol Metab. 2010;298(2):E257-E69.
11. Hulston CJ, Woods RM, Dewhurst-Trigg R, Parry SA, Gagnon S, Baker L, et al. Resistance exercise stimulates mixed muscle protein synthesis in lean and obese young adults. Phys Rep. 2018;6(14):11.
12. Kim PL, Staron RS, Phillips SM. Fasted-state skeletal muscle protein synthesis after resistance exercise is altered with training. J Physiol. 2005;568(1):283-90.
13. Kumar V, Atherton PJ, Selby A, Rankin D, Williams J, Smith K, et al. Muscle protein synthetic responses to exercise: effects of age, volume, and intensity. J Gerontol A Biol Sci Med Sci. 2012;67(11):1170-7.
14. Kumar V, Selby A, Rankin D, Patel R, Atherton P, Hildebrandt W, et al. Age‐related differences in the dose–response relationship of muscle protein synthesis to resistance exercise in young and old men. J Physiol. 2009;587(1):211-7.
15. Phillips SM, Tipton KD. Resistance training reduces the acute exercise-induced increase in muscle protein turnover. Am J Physiol. 1999;276(1):E118.
16. Phillips SM, Tipton KD, Aarsland A, Wolf SE, Wolfe RR. Mixed muscle protein synthesis and breakdown after resistance exercise in humans. Am J Physiol Endocrinol Metab. 1997;273(1):E99-E107.
17. Robinson MJ, Burd NA, Breen L, Rerecich T, Yang Y, Hector AJ, et al. Dose-dependent responses of myofibrillar protein synthesis with beef ingestion are enhanced with resistance exercise in middle-aged men. Appl Physiol Nutr Metab. 2013;38(2):120-5.
18. Sheffield-Moore M, Paddon-Jones D, Sanford A, Rosenblatt J, Matlock A, Cree M, et al. Mixed muscle and hepatic derived plasma protein metabolism is differentially regulated in older and younger men following resistance exercise. Am J Physiol Endocrinol Metab. 2005;288(5):E922-E9.
19. Trappe TA, Raue U, Tesch PA. Human soleus muscle protein synthesis following resistance exercise. Acta Physiol Scand. 2004;182(2):189-96.
20. Trappe RA, White F, Lambert CP, Cesar D, Hellerstein M, Evans WJ. Effect of ibuprofen and acetaminophen on postexercise muscle protein synthesis. Am J Physiol Endocrinol Metab. 2002;282(3):E551-E6.
21. Yang Y, Churchward-Venne TA, Burd NA, Breen L, Tarnopolsky MA, Phillips SM. Myofibrillar protein synthesis following ingestion of soy protein isolate at rest and after resistance exercise in elderly men. Nutr Metab. 2012;9:9.
